# Rheumatoid cachexia: the underappreciated role of myoblast, macrophage and fibroblast interplay in the skeletal muscle niche

**DOI:** 10.1186/s12929-021-00714-w

**Published:** 2021-03-03

**Authors:** T. Ollewagen, K. H. Myburgh, M. van de Vyver, C. Smith

**Affiliations:** 1 Department of Physiological Sciences, Science Faculty, Stellenbosch University, Stellenbosch, South Africa; 2 Division of Clinical Pharmacology, Department of Medicine, Faculty of Medicine and Health Sciences, Stellenbosch University, Parow, South Africa

**Keywords:** Intracellular communication, Arthritis, Co-culture, Macrophage, M2b, Extracellular matrix

## Abstract

Although rheumatoid arthritis affects 1% of the global population, the role of rheumatoid cachexia, which occurs in up to a third of patients, is relatively neglected as research focus, despite its significant contribution to decreased quality of life in patients. A better understanding of the cellular and molecular processes involved in rheumatoid cachexia, as well as its potential treatment, is dependent on elucidation of the intricate interactions of the cells involved, such as myoblasts, fibroblasts and macrophages. Persistent RA-associated inflammation results in a relative depletion of the capacity for regeneration and repair in the satellite cell niche. The repair that does proceed is suboptimal due to dysregulated communication from the other cellular role players in this multi-cellular environment. This includes the incomplete switch in macrophage phenotype resulting in a lingering pro-inflammatory state within the tissues, as well as fibroblast-associated dysregulation of the dynamic control of the extracellular matrix. Additional to this endogenous dysregulation, some treatment strategies for RA may exacerbate muscle wasting and no multi-cell investigation has been done in this context. This review summarizes the most recent literature characterising clinical RA cachexia and links these features to the roles of and complex communication between multiple cellular contributors in the muscle niche, highlighting the importance of a targeted approach to therapeutic intervention.

## Introduction

Rheumatoid arthritis (RA) is a multifactorial disease that affects approximately 1% of the global population. Whilst the exact aetiology of RA is still being elucidated, it is known that both genetic and environmental factors initiate the auto-immune response against the synovium by stimulating several cell types and their cytokine secretome [1, 2].

RA targets not only the joints, but also the surrounding tissues and their resident and infiltrating cellular components resulting in disability and impaired quality of life [3, 4]. In this context, a recent review [5] highlighted the underappreciated status of skeletal muscle and emphasized the significant role of muscle health in the preventing disability and cardiometabolic disease. In RA patients there is a reported 7.4–14.0% decrease in muscle mass compared to matched controls, which frequently occurs with a concomitant increase in fat mass [3]. A recent meta-analysis indicated the presence of rheumatoid cachexia in 15–32% of RA cases [6]. Cachexia is defined as a “complex metabolic syndrome associated with underlying illness and characterized by loss of muscle with or without loss of fat mass” [7]. Cachexia can be diagnosed through the assessment of body composition with either bioimpedance analysis or dual energy X-ray absorptiometry (DEXA) [8]. It may be beneficial to consider additional factors for diagnosis including: anorexia, inflammation, metabolic disturbances, physical output, and quality of life [9].The exact molecular mechanisms involved in rheumatoid cachexia are still unclear, but the most popular hypotheses include reduced physical activity associated with joint pain, pro-inflammatory cytokine-induced catabolism, insulin resistance, insufficient protein ingestion and reduced anabolism [4, 10]. It is also possible that these different mechanisms occur concomitantly or separately during the different phases of disease. These hypotheses are, in part, based on the links between inflammation, muscle loss and dysfunction that have been elucidated in other diseases as diverse as diabetes and cancer [11–14]. Although rheumatoid cachexia may share some of the mechanisms, the nature of auto-immune diseases increases the complexity of the inflammatory milieu. Another factor to consider is that which neuroinflammation associated with RA presents prior to joint destruction, and the potential effect this may have muscle innervation and the loss of mass and strength, although not sufficiently investigated [15, 16]. The early onset of inflammation opens the window for earlier treatment targeted at specific mechanisms of rheumatoid cachexia. Earlier intervention could feasibly delay the point where dysregulation is so severe that cachexia becomes clinically evident. It is therefore imperative to determine the causative mechanisms involved in rheumatoid cachexia, to provide the basis for the development of early diagnostic predictors, as well as new preventative and therapeutic strategies targeting the regulatory mechanisms.

A limitation of studies investigating the dysregulation of different cellular role players in rheumatoid cachexia, is that they neglect the complex interaction by focussing on one particular cell type in isolation. Although useful in terms of assessing cellular dysfunction, this does not provide a complete picture of rheumatoid cachexia aetiology. Skeletal muscle is a complex tissue niche, containing several co-existing resident cell types, including myoblasts, fibroblasts and a variety of immune cells, which affect each other multi-directionally via various molecular communication mechanisms.

Due to alterations in the relative distribution and type of immune and cells fibroblasts in different myopathies [17], a comprehensive, integrative assessment of their availability and intercellular signalling in RA is vital to form a holistic picture of dysregulation and/or muscle pathology. As RA is an auto-immune disease, its inflammatory profile is somewhat unique when compared to other chronic inflammatory diseases [18], so that mere suppression of inflammation—either in circulation or in the synovia—may not be sufficient to prevent RA-associated detrimental effects on skeletal muscle health. Additionally, inflammation contributes to muscle repair processes and pure suppression would therefore impair regeneration [19], as discussed in later sections. Thus, a more comprehensive, integrated understanding of the different role players involved in rheumatoid cachexia is required to further develop treatment strategies.

This review will provide a brief overview of the known dysregulation and clinical manifestation of rheumatoid cachexia, as well as potential aetiological role players. It is followed by a discussion focussed on integrating the roles of the different cell types present in the muscle niche and their paracrine communication in the context of RA-associated inflammation and muscle de- and regeneration.

### The clinical features of rheumatoid cachexia within skeletal muscle

The centrally reported feature of rheumatoid cachexia is the presence of increased levels of pro-inflammatory cytokines (systemic and locally), tumour necrosis factor-α (TNF-α) and interleukin-1β (IL-1β) [6]—which are also primarily targeted in current treatment modalities. In skeletal muscle, inflammation affects normal protein turnover as well as its response to injury. Both processes involve coordinated remodelling of skeletal muscle tissue with the latter involving the activation, proliferation and differentiation of satellite cells—muscle-specific stem cells [20].

Skeletal muscle mass is easily influenced both positively and negatively, either through hypertrophy or atrophy, both of which are facilitated by signalling pathways involved in protein turnover [21]. Hypertrophy of the muscle occurs in response to growth stimuli and an increase in protein synthesis, while muscle atrophy occurs with several pathological states including chronic inflammation, disuse and ageing. Cachexia is a well-known consequence of a number of chronic inflammatory diseases including cancer, chronic obstructive pulmonary disease (COPD), chronic heart disease (CHD), cystic fibrosis and RA, amongst others [22]. However, the functional effects of the associated muscle atrophy are underestimated, especially since rheumatoid cachexia exhibits both reduced muscle mass and muscle performance, which may lead to disability, poor quality of life and increased mortality.

The specific aetiological role players that determine rheumatoid cachexia outcome are summarised next.

### Clinical disease severity correlates with loss of skeletal muscle fibre size

Cross-sectional area (CSA) is indicative of the potential for muscle force output which is affected not only by muscle size, but also density, fascicle length and the angle of pennation. Several studies in RA patients and animal models of RA report that RA is characterised by a parallel loss of CSA and strength. An early study reported a simple linear regression between grip strength and muscle CSA for both healthy and RA individuals [23]. In line with this, a rodent collagen-induced arthritis (CIA) model indicated a strong correlation between clinical disease scores (arthritis severity in peripheral joints) and locomotion, which was associated with a significant loss in muscle mass across multiple muscle types (tibialis anterior (TA), extensor digitorum longus (EDL), soleus and gastrocnemius muscles) [24]. Similarly, another rodent study using the same CIA model demonstrated reduced myofibre CSA of gastrocnemius and TA muscles alongside severe arthritic changes including extensive bone erosion and cartilage thinning, within 45 days of onset. In this study, a significant inverse correlation was reported between disease clinical scores and myofibre CSA, highlighting the relationship between disease severity and degree of atrophy [4]. Based on these studies, it is clear that a reduction in CSA occurs as a result of RA and is one of the key identifying factors of rheumatoid cachexia.

### Cachexia profile is time-dependent and not dependent on pain-associated inactivity

Initially, it was suggested that the atrophy associated with RA occurred as a result of inactivity due to painful joints. However, it has since been observed that muscle atrophy and weakness are already present before pain and swelling affect the physical activity of the RA patient [25]. For example, a reduction in several performance outcomes such as knee extension, grip and trunk extension strengths, as well as overall muscle strength index (29%), were reported in RA patients when compared to controls, at a time point early after clinical onset of RA, and preceding bone mineral density loss [26]. Patients with stable RA and uncompromised appendicular lean mass exhibited significantly higher body fat percentage and significantly *smaller vastus lateralis* muscle CSA than matched controls, as well as a reduction in objective physical function [27]. This suggests that CSA is already affected from early stage disease and is not rescued even when patients reach a stable state. However, in another report, a reduction in CSA did not affect contractile properties, activation capacity and concentric force, or power elicited by electrical stimulation—a finding that may indicate some loss in voluntary activation due to pain. Alternatively, since pennation angle was reduced with atrophy, this might explain an improved direction of force transduction with maximal stimulation despite reduced CSA [27]. Taken together, these data support the conclusion that skeletal muscle CSA is altered early in disease progression and contributes to loss of function in RA patients. This suggests that at least some compensatory muscle regenerative mechanisms may be activated with time, to counter early cachexia-associated loss of strength. These events at cellular levels remain to be fully elucidated as will be discussed later in this review.

### Body composition contributes to the severity of cachexia-induced muscle function deficit

Another potential determining factor in the reduction of muscle strength is the accumulation of intramuscular fat (as indicated by decreased muscle density on quantitative computer tomography (QCT) scans). Low muscle density is indicative of increased myocellular lipid content and fatty infiltration in the interstitial space and is a strong negative indicator of muscle quality. In this context, a cross-sectional study on 60 RA patients indicated associations between a low skeletal muscle fat mass (within individual muscles; includes fat inside myocytes and around the muscle fibres) and more effective performance, including greater speed and quadriceps muscle strength [28]. Similarly, excessive total body fat (obesity) seems to also be a contributing factor in the decline of muscle function. Kramer et al*.* (2012) demonstrated an inverse relationship between muscle density and body fat mass in the context of RA. The same study reported a correlation between muscle density decline and numerous factors related to the inflammatory profile such as increased age, RA duration, IL-6 and TNF-α levels, as well as circulating endogenous glucocorticoids [29]. Taken together, these data illustrate the important role of inflammatory activation in determining muscle function and highlight that the combination of metabolic and inflammatory dysregulation adds to the complexity of the disease in RA patients.

### The role of skeletal muscle fibre type composition

In terms of muscle type-specific sensitivity to rheumatoid cachexia, reports are varied. Generally, changes in catabolic responses between predominantly glycolytic or oxidative skeletal muscle types have been assessed in several non-RA models of chronic inflammation including burn-injury [30], chronic heart failure [31], and sepsis [32] with data suggesting that fast-twitch glycolytic muscles are more prone to atrophy. In RA specifically, early patient-based studies have reported the most severe atrophy in type II fibres from the quadriceps femoris muscle [33, 34]. Of interest to the current review topic, some also reported increased collagen fibril deposition around fibres exhibiting mild necrosis (but not in fibres with severe atrophy or necrosis) [34]. This is in line with our earlier suggestion that cachectic muscle may activate regenerative counter-mechanisms.

Recent animal studies utilizing the CIA model are however more contradictory. On one hand, assessing the gastrocnemius as a predominantly fast-twitch and the soleus as a predominantly slow-twitch muscle, the loss of muscle mass in rats was more pronounced in the gastrocnemius than the soleus [35]. Similarly, in female cynomolgus monkeys, irregularity in muscle fibre size and more prominent atrophy in type II muscle fibres 5 weeks after initial immunization was demonstrated [36]. In contrast, no difference in muscle weight and force production deficits between soleus and EDL muscles were reported in mice, suggesting that changes in gastrocnemius and EDL muscles might differ [37]. However, a recent study done by our group assessing the gastrocnemius, soleus and EDL muscles in rats, demonstrated a severe (≈60%) loss of muscle mass, but with similar severity in terms of reduction in CSA, left shift of fibre size distribution and histological evidence of fibre degradation [38], supporting the argument against muscle fibre type as major role player. Interestingly, atrophy was less pronounced in the vastus lateralis muscle, which suggests that other factors, such as body posture, distance of muscle from affected joints, or even muscle-specific differences in redox profile—which was detailed in this study [34]—may contribute relatively more to final outcome than fibre type itself.

The next sections will provide a more in-depth explanation of the complex signalling from multiple cellular role players contributing to rheumatoid cachexia.

### Proinflammatory cytokine-induced protein degradation and catabolism in rheumatoid cachexia

**Fig. 1:**
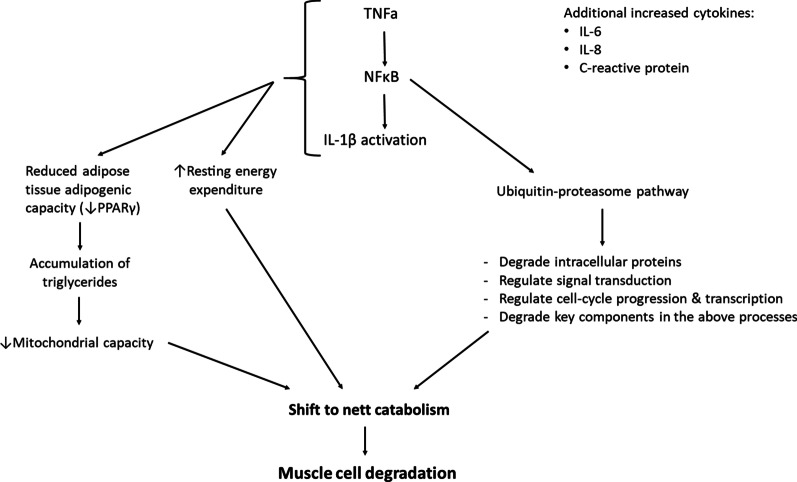
Pathways of proinflammatory cytokine-induced protein degradation in rheumatoid cachexia. (ref [35, 40–45]). *TNFα* tumor necrosis factor-α, *NFκB*  nuclear factor kappa-light chain enhancer of activated B cells, *IL-1β*  interleukin-1β, *PPARγ*  peroxisome proliferator activated receptor-gamma

Skeletal muscle is a relatively adaptive tissue and is composed of muscle fibres connected within the extracellular matrix (ECM). Skeletal muscle cells are particularly protein rich and the normally encountered mechanical strains require a basal protein turnover to enable maintenance of structural and functional proteins, adjustments to important regulatory functions and a plethora of adaptive responses. It is important that this process of protein turnover remains balanced, as a small increase in degradation or a decrease in synthesis can result in a reduction in overall cell mass which ultimately leads to muscle atrophy [10]. As depicted in Fig. 1, systemic inflammation promotes protein degradation which leads to the loss of body cell mass, and unfortunately targets predominantly lean muscle tissue [19, 39–43].

Taken together, these studies again indicate that the increase in protein catabolism is largely associated with an increased presence of pro-inflammatory cytokines, which ultimately results in rheumatoid cachexia. The fact that elevated cytokine expression no doubt occurs prior to clinical manifestation of RA joint symptoms, also explains why the loss of muscle mass is already observed in patients early after clinical onset of RA.

### Cellular mediators and molecular dysregulation giving rise to rheumatoid cachexia

Characterisation of muscle pathology in rheumatoid cachexia has gradually progressed since microscopic assessments of different muscle groups in 100 RA patients first revealed muscle fibre atrophy, abundant fibrinoid material and immune cells over 40 years ago [44]. A decade after this first report, the muscle pathology associated with RA was determined to be more complex and was labelled rheumatoid myositis (RM). This was based on electron microscopy evidence of necrosis, varied degrees of atrophy, infiltration of mononuclear cells and myopathic changes. Other reported features included fewer and widely separated myofibrils, the presence of more collagen fibrils (on the myofibre surface and in extracellular spaces) as well as disorientated myofilaments [34]. These early papers already highlighted the involvement of multiple different cell types in RA pathology. More recent studies further indicate that it is possible for patients with RA to show signs of muscle regeneration. For example, Boutrup and colleagues recently demonstrated that higher myonuclear content in muscle fibres in *vastus lateralis* muscle biopsies taken from RA patients, when compared to healthy controls [45]. This suggests that donation of additional nuclei from the satellite cell pool occurred as a possible compensatory mechanism to preserve muscle health/function.

Below, we review the most relevant literature on relevant cellular role players and their molecular interactions. For clarity of the argument, interactions between skeletal muscle and inflammatory cells are discussed first, in terms of the balance between tissue de- and regeneration. This is followed by a literature overview and discussion of the interplay of fibroblasts and fibrosis in terms of quality of muscle repair.

### Satellite cell activation and cell–cell cross-talk in response to inflammation and during regeneration and regrowth

**Fig. 2:**
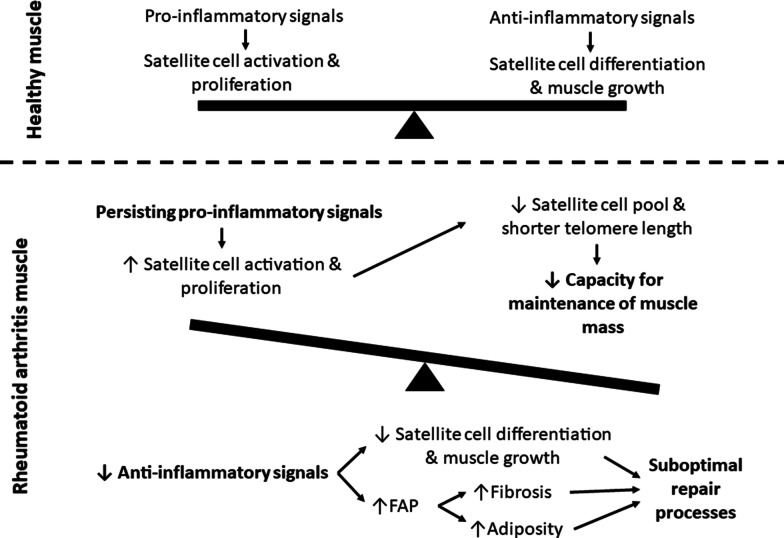
The imbalance between pro- and anti-inflammatory signalling in RA rodent skeletal muscle, and the resulting influence on muscle repair and growth. *FAP*  fibro-adipogenic progenitor cell

Skeletal muscle has a unique ability to regenerate throughout life due to the presence of satellite cells. Satellite cells reside between the basal lamina and the sarcolemma and in close proximity to capillaries [46], thus poised to be influenced by the mature muscle cells (fibres), ECM, other cells in the niche and circulating factors. Under healthy, resting conditions, the satellite cells remain quiescent. However, these cells play a major role following injury, at which time they are rapidly activated to proliferate, differentiate and fuse in order to repair the damaged area [47]. Mechanical loading of muscle with chronic underlying pathology leads to acute damage [48]. Hence, skeletal muscle may be required to go through episodic regeneration. Over the past decade, another resident interstitial mesenchymal-like stem cell, the fibro-adipogenic progenitor (FAP) cell, has been identified as important in regulating satellite cells [49] via engaging in communication with other cells in the satellite cell niche [50]. These include immune cells and fibroblasts which play critical roles in all the stages of muscle repair [51]. Figure 2 summarises the relationship between the different cell type influencing muscle growth and repair in inflammatory conditions such as rheumatoid arthritis [34, 49–65].

In a chronic inflammatory setting such as RA, the persistent imbalance of pro- and anti-inflammatory macrophages disturbs normal muscle regeneration by impairing satellite cell proliferation and differentiation [51, 55, 56]. It is important that the macrophage subtypes work together to maintain the balance between proliferating and differentiating satellite cells. In a rodent model of RA, an imbalance between M1 and M2 macrophages favouring excess M1, has been shown to be related to continued invasion of monocytes into synovial tissue [66]. Similarly, macrophage inflitration perpetuates damage in chronic muscle pathologies [67]. It is conceivable that this occurs in the muscle of RA patients, even though the muscle damage is a secondary pathology. Given this evidence of the involvement of macrophages and/or their secreted cytokines as critical contributors to the maintenance of skeletal muscle integrity, a dysregulatory shift in macrophage phenotype can significantly alter the delicate balance between proliferation, differentiation, and fusion of myoblasts to repair or maintain the muscle fibres. In order to better understand the feasibility of therapeutically targeting macrophages in RA, it is necessary to better understand the different immune cell types involved.

### A more in-depth review of dysregulated macrophage polarization in RA

In joints with RA, the synovial membrane is also complex in terms of cellular profile, containing activated B- and T-cells, plasma cells, mast cells, and monocytes. Additionally, the presence of activated synovial fibroblasts, chondrocytes, and osteoclasts contributes to cartilage and bone destruction and continued inflammation through the sustained release of cytokines [68]. These cell types are not limited to the synovial joint and can be found across numerous tissue types, including skeletal muscle [55]. Maladaptation, disease-associated pathology or therapeutic interference with the inflammatory response can affect the possibility of synovial joint and muscle repair and long-term function significantly. When targeting macrophages for therapeutic effect, the phenotype plasticity of macrophages complicates matters. Temporary ablation of macrophages/monocytes through diphtheria toxin injection after cardiotoxin injury in the TA muscle of mice was reported to attenuate the phenotypic switch of macrophage subsets and ultimately impair muscle regeneration [69]. Conversely, co-injection of pro-inflammatory macrophages with myoblasts into regenerating mouse skeletal muscle resulted in the generation of twice as many fibres after 4 weeks compared to myoblasts alone or myoblasts with anti-inflammatory macrophages in vivo. In this study, subsequent co-culture of myoblasts and macrophages illustrated that co-culture with pro-inflammatory M1 macrophages resulted in an increase in the number of proliferating myoblasts and a decrease in differentiation. The opposite was observed with anti-inflammatory M2 macrophage co-culture [19]. This indicates that while persistent inflammation can be detrimental to muscle, and despite the opposing roles of M1 and M2 macrophages, the synchronised presence of both phenotypes is vital for optimal skeletal muscle regeneration.

Pro-inflammatory M1 macrophages arise in response to IFN-γ, TNF-α, granulocyte–macrophage colony-stimulating factor (GM-CSF) and lipopolysaccharide (LPS)/endotoxin in the early stages of muscle repair. Their major role is the removal of necrotic material and to process and present antigens to activate T-cells. M1 macrophages produce pro-inflammatory cytokines TNF-α and IL-1β. The M2 macrophage population is more complex and can be divided into four subtypes, namely M2a, b, c, and d. M2a macrophages are activated by IL-4 and IL-13 and contribute to tissue repair, wound healing and fibrosis in most scenarios of muscle damage. M2a macrophages are most abundant in the later stages of muscle repair [54, 57] and contribute to fibrosis through the secretion of transforming growth factor (TGF)-β, insulin-like growth factor (IGF) and fibronectin.

In acute damage scenarios, M2a macrophages will switch to M2c macrophages once the inflammatory stimulus is abolished, due to a change in the intracellular cytokine profile. Specifically, M2c macrophages are activated by IL-10 and TGF-β and release anti-inflammatory cytokines, in particular IL-10, to resolve inflammation and limit fibrosis [70]. However, in scenarios of prolonged or chronic inflammation, M2b macrophages are predominantly found. Various signalling factors are involved in the polarisation of macrophages towards a predominance of M2b macrophages, including NF-κB, mitogen-activated protein kinases (MAPKs), PI3K/Akt and interferon regulatory factors (IRFs). M2b macrophages display the capacity to secrete both pro-inflammatory cytokines such as TNF-α, IL-1β and IL-6, and anti-inflammatory cytokines such as IL-10 [71, 72], but the factors determining the nett profile of their secretomes are less clear. M2b macrophages are thought to have a role in limiting fibrosis after *acute* cardiac muscle injury [72]. Recently, micro-RNA (miRNA)-125a-5p overexpression [73] and chemokine (C–C motif) ligand 1 (CCL1) signalling [74] was however implicated in the M2b polarisation seen in juvenile idiopathic arthritis which is associated with poor resolution of inflammation.

**Fig. 3:**
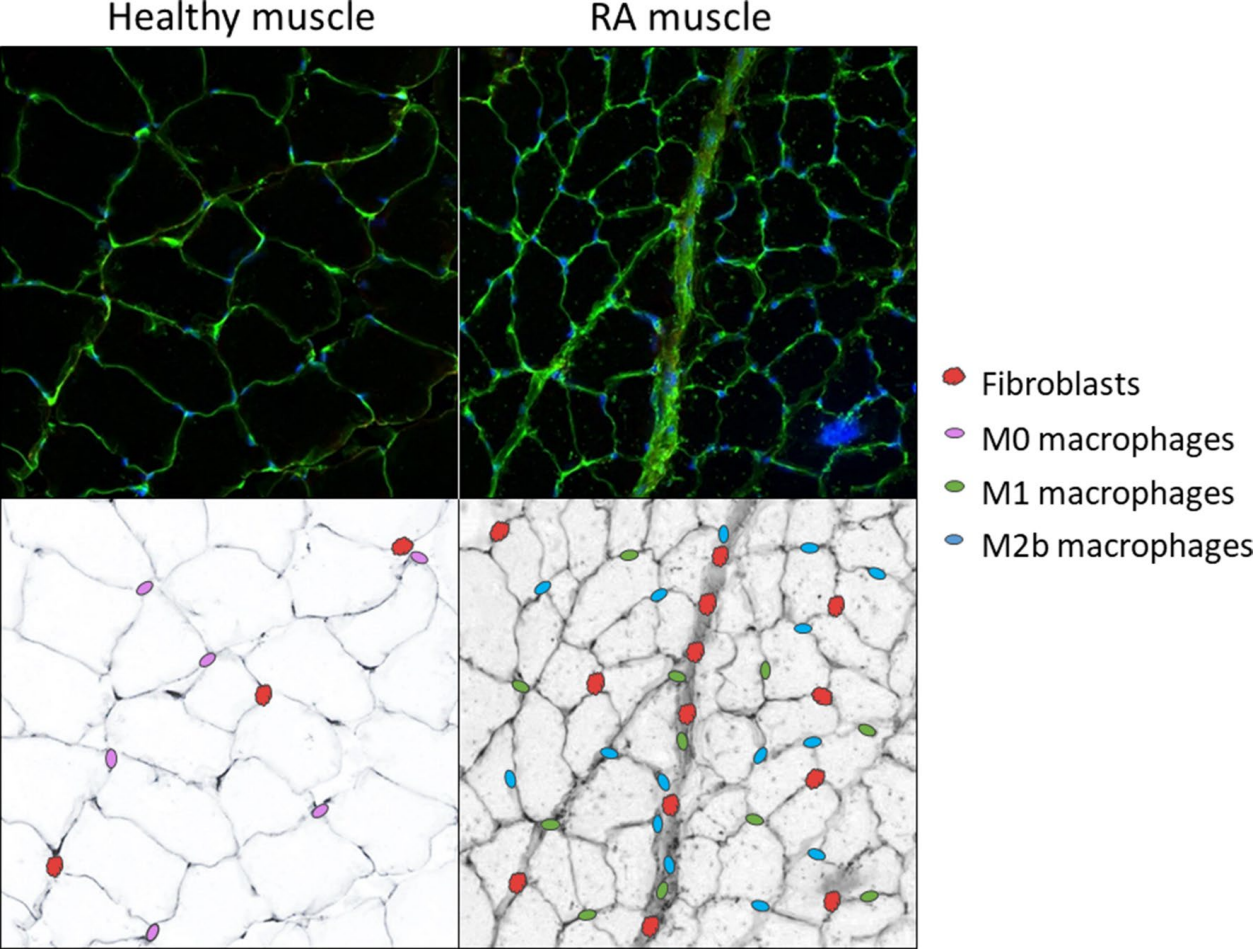
Representative images suggesting the presence of different cell types in healthy versus rheumatoid arthritis skeletal muscle. Fluorescent images from a RA rodent model (study execution described in Oyenihi et al., 2019 [38]; staining method described in Additional file 1) indicate clear cachexia and increased fibrosis between muscle fibres. Black and white images indicative of the authors prediction of greater presence of macrophages (M1 and M2b) and fibroblasts in RA

Studies suggest that RA patients exhibit persistence of more pro-inflammatory macrophage phenotypes when compared to patients with peripheral spondylarthritis [75] and osteoarthritis [76], where a shift towards an M2 phenotype has been reported. Macrophage depletion using clodronate-containing liposomes prior to initiation of arthritis in a CIA rodent model resulted in reduced M1 cell presence in the joint, alongside a reduction in arthritis disease score, indicating the importance of M1 macrophages in the development of RA [77]. However, further investigation is required to fully understand the significance of the M2 subset shifts in RA aetiology. Nonetheless, it would seem that in chronic inflammatory disease, the natural phenotype shift—which occurs via cytokine-induced polarisation—is skewed to limit the complete phenotype transition to achieve anti-inflammatory M2c macrophages (Fig. 3). The extracellular signalling factors responsible for this phenomenon, are ideal candidates for therapeutic targeting. (The M2d phenotype is associated with angiogenesis in tumour growth and not relevant to the current topic.)

### Fibroblasts and the extracellular matrix (ECM)

During the regeneration and remodelling phases after muscle injury, structural aspects of muscle are in flux and hence muscle quality may be reduced. Fibroblasts secrete several growth factors and ECM components (fibronectin, collagen I and III, and proteoglycans) during maintenance and repair. Following damage, binding of ECM components (such as fibrin and fibronectin) to the collagens and proteoglycans form a temporary matrix within the injury site. This provides a suitable environment for differentiating myoblasts and a scaffold for regenerating myofibres [78–80].

Myofibroblasts contribute to tissue repair through wound contracture but may also play a role in the formation of fibrosis. Myofibroblasts were originally thought to be derived only from differentiating resident fibroblasts, however, they can also arise from parenchymal epithelial cells through epithelial to mesenchymal transition (EMT) [81, 82] and from FAPs [83]. For optimal repair, these cells are present in damaged muscle only transiently, with a rapid reduction in their numbers in the regenerating area. However, in chronic situations, the persistence of FAPs and their differentiation into myofibroblasts and adipocytes results in intramuscular fibrofatty infiltration [50, 83]. Here, macrophage dysregulation comes into play: the increase in TGF-β secretion by M2 macrophages blocks the TNF-α-induced apoptosis, resulting in prolonged FAP survival. Although not directly assessed in RA, the increased muscle fat mass reported in RA suggest that these processes also contribute to RA myopathy.

Degradation of the temporary ECM occurs after sufficient repair to allow for the optimal growth of the regenerating fibres [65, 81]. Matrix metalloproteinases (MMPs) produced by damaged myofibres and infiltrating cells play a key role in the degradation of this temporary ECM [50, 84]. However, the excessive deposition of ECM and impaired degradation via TGF-β-dependent mechanisms leads to interstitial fibrosis and subsequent loss of tissue architecture and function. Within the skeletal muscle, excessive ECM deposition, especially collagens, results in impaired muscle fibre regeneration and increased susceptibility to re-injury, ultimately causing morbidity and mortality [81, 82].

**Fig. 4:**
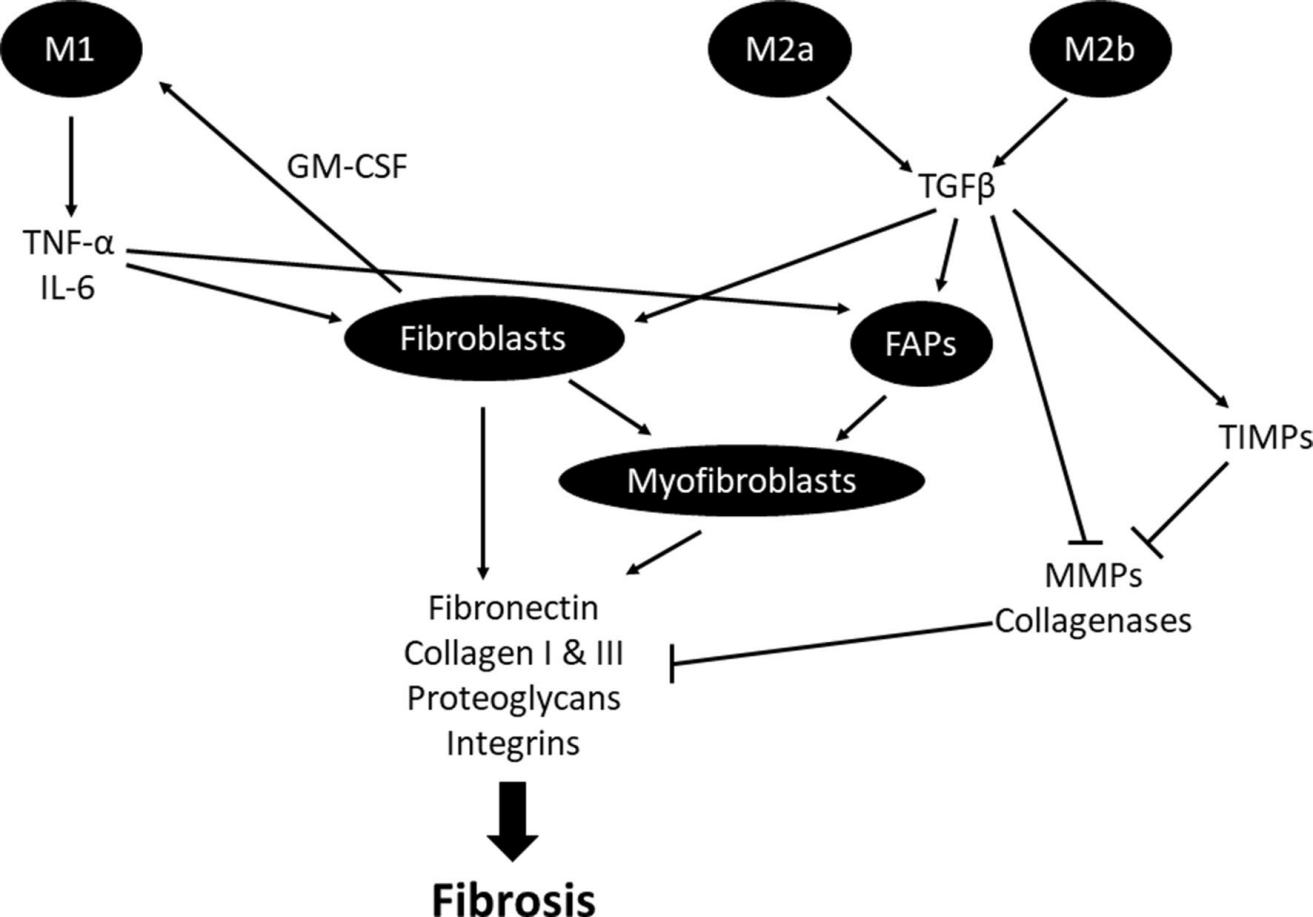
Summary of the interaction between macrophages, fibroblasts and FAPs in the development of tissue fibrosis. *TNF-α*  tumor necrosis factor-alpha, *IL-6*  interleukin-6, *TGFβ*  transforming growth factor-beta, *FAPs*  fibro-adipogenic progenitor cells, *TIMPs*  tissue inhibitor of metalloproteinase, *MMPs*  metalloproteinases

Macrophages are attracted to the damaged tissue by chemoattractant cytokines and secrete cytokines that indirectly stimulate the production of ECM components, as summarized in Fig. 4. M1 macrophages release TNF-α and IL-6 which stimulate the proliferation of fibroblasts and FAPs [85], but upon binding to excess fibrinogen also increase the production of IL-1β and TGF-β [86]. TNF-α and IL-1β increase the synthesis of collagenases contributing to the destruction of the cartilage in RA [45, 53, 56]. M2a macrophages play a profound role in fibrosis due to the fact that they release a large range of pro-inflammatory molecules such as TGF-β, fibronectin, several TIMPs and CCL17 [56, 82]. In addition, both CCL3 (macrophage inflammatory protein 1α) and CCL2 (monocyte chemoattractant protein-1) have been highlighted as promotors of fibrosis through their chemotactic properties [81]. To add to this, fibroblasts were shown to have the capacity to promote arthritis through the production of GM-CSF, which enhances the survival of neutrophils and macrophages [87], thus further strengthening the inflammatory response in a vicious cycle, much like the self-propagating oxidative damage-inflammation cycle that also comes into play in chronic diseases such as RA [38]. Disturbance in the balance between classically activated (M1) and alternative (M2) macrophages therefore leads to excessive TGF-β production, resulting in excessive activation of fibroblasts and inhibited apoptosis of FAPs, resulting in excessive ECM production and fibrosis [56, 80]. Related to this, it was suggested by Khoja [28] that the accumulation of fat around the muscle spindles may lead to a similar effect with thickening of the capsule and fibrotic changes in the intrafusal muscle fibres. This has not yet been sufficiently assessed in the context of RA, warranting further research in this context.

One of the most important factors involved in tissue healing and fibrosis is TGFβ. This profibrotic growth factor is present in skeletal muscle following injury and in dystrophic muscle, where it stimulates fibroblasts to produce ECM components [79, 88]. During regeneration, the degradation of the initial scaffold of ECM contributes to the generation of protein fragments that mediate biological activities involved in normal tissue repair [73, 79, 85]. However, TGFβ reduces the production of enzymes (such as collagenase) and stimulates the production of tissue inhibitors of metalloproteinases (TIMPs) and plasminogen activator inhibitor type-1 (PAI-1), thereby inhibiting the degradation of the ECM [83, 88] and disturbing the final outcome.

Gene expression analysis of skeletal muscle samples from RA patients demonstrated a correlation between disease activity and disability and increased concentrations of amino acid precursors to muscle fibrosis [20]. Analysis of blood samples from patients with active RA highlighted differences in cell–cell interactions, altered EMT, and increased TGF-β proteins suggestive of increased de novo ECM synthesis and fibrosis [89]. Fibrosis has been demonstrated in several tissues from RA patients and rodent studies. For example, increased fibrous tissue deposition was demonstrated in the joint of RA rodents 6 days post induction with fluorescent visualization of collagen III and fibrinogen [90]. Similarly, research from our group has demonstrated a significant increase in collagen accumulation within the vastus lateralis, soleus and gastrocnemius muscles in CIA rodents compared to that of control rodents [38], as well as impaired molecular remodelling with fibrotic deposition and impaired cardiomyofibre contractile function [91]. In humans, the presence of subclinical myocardial fibrosis resulting from low grade chronic inflammation in a large number of RA patients, has been linked to heart failure in approximately 3.9% of patients [92]. Furthermore, pulmonary fibrosis is often associated with RA as a result of increased TGF-β and Smad signalling leading to an increase in collagen deposition within the lung tissue [93]. The assessment of fibrosis in RA skeletal muscle tissue is limited, however in chronic inflammation, macrophages expressing both pro- and anti-inflammatory cytokines (TNFα and TGFβ) were reported to have a reduced ability to clear FAPs from the damaged tissue [50]. Cross-talk between these cells and satellite cells also occurs via TGFβ. In vitro, it has been demonstrated that the isoform of TGFβ has an influence on the satellite cell response [94]. Therefore, a full understanding of the role of TGFβ in RA may require that level of sophistication, which can be achieved in vitro in single cell culture or co-culture. In chronic kidney disease, chronic inflammation resulted in increased muscle collagen content which also correlated with increased abundance of FAPs [95].

To further complicate matters, in vitro studies have demonstrated that satellite cells themselves also secrete factors that regulate ECM gene expression independent of TGF-β [96, 97]. The use of Pax7 knock-out mice indicated that satellite cells are critical to limit ECM deposition and prevent fibrosis in the first week of regeneration, potentially through a mechanism involving microRNA and exosomes circulating the skeletal muscle. For example, microRNA-206 (miR-206) is highly expressed in satellite cells and satellite cell-derived exosomes; it performs its actions through binding to and inhibiting ribosomal binding protein 1 (Rrbp1), a master regulator of collagen synthesis. Knockdown of miR-206 resulted in the increased expression of collagen genes in fibrogenic cells [97], proposing a potential mechanism of fibrogenic pathogenesis in chronic inflammatory disorders such as RA. The potential for therapeutic or preventative intervention at this level warrants further investigation.

### Finding therapeutic targets in the context of RA

From the literature presented above, it is clear that complex, aberrant intracellular communication resulting from auto-immune activation—and thus persistent inflammation—results in the complex syndrome of rheumatoid cachexia.

**Fig. 5:**
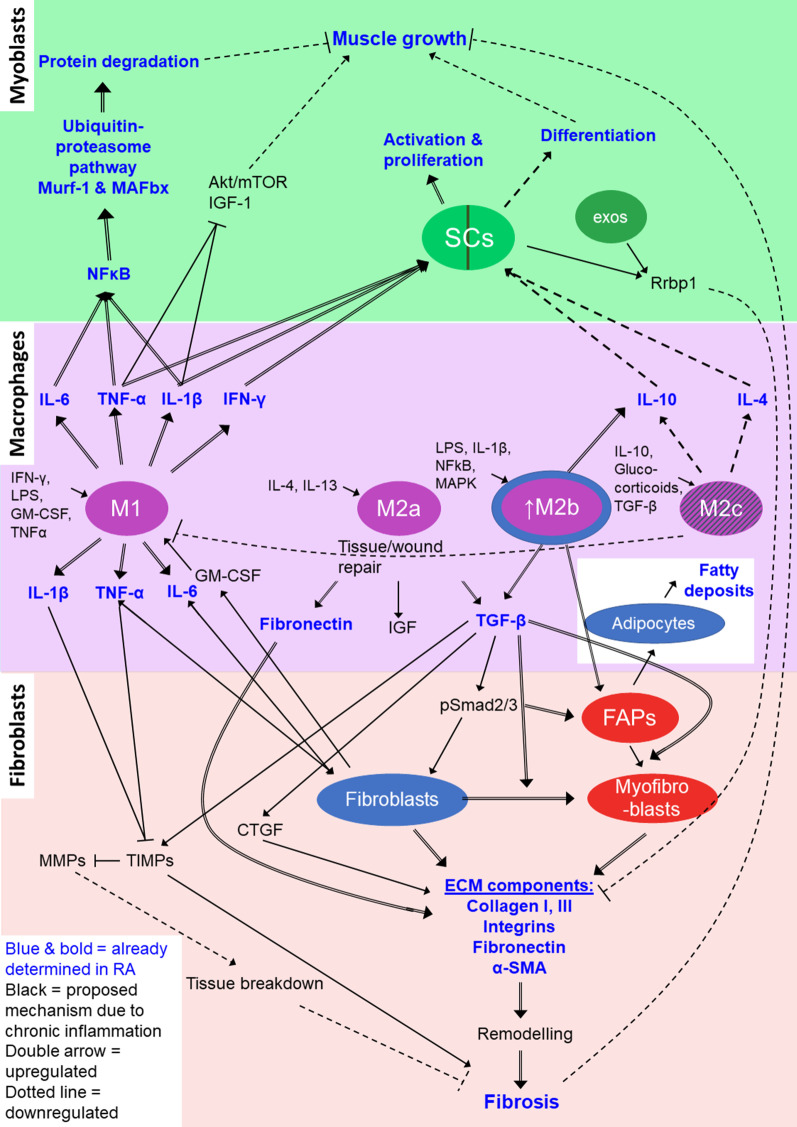
Summary of the cellular interactions in chronic inflammation leading to a decline in muscle growth. The incomplete switch from M1 to M2c results in a greater presence of M2b macrophages which present both impaired pro- and impaired anti-inflammatory properties, often resulting in enhanced deposition of ECM components, and impaired satellite cell function. This also results in a reduced presence of M2c macrophages. The mechanisms and pathways presented are based on chronic inflammatory microenvironments (proposed in RA), with blue indicating mechanisms confirmed in studies of RA. Different colours indicate different cell focus areas; green = muscle niche; purple = inflammatory system; red = fibroblasts and fibrosis. Increased signalling are indicated by double-line arrows, while dotted line arrows indicate decreased signalling. *SCs*  satellite cells, *IGF*  insulin-like growth factor, *Murf-1*  muscle ring finger protein-1, *Mafbx*  muscle atrophy f-box, *exos*  exosomes, *Rrbp1*  ribosome binding protein-1, *IL*  interleukin, *TNF-α*  tumor necrosis factor-α, *IFN-γ* interferon-γ, *LPS*  lipopolysaccharides, *GM-CSF*  granulocyte-monocyte colony-stimulating factor, *TGF-β*  transforming growth factor-β, *TIMP*  tissue inhibitor of metalloproteinase, *MMP*  matrix metalloproteinase, *CTGF*  connective tissue growth factor, *FAPs*  fibro-adipogenic progenitor cells, *ECM*  extracellular matrix, *α-SMA*  α-smooth muscle actin

We present a predictive image of the muscle niche in Fig. 3 and a visual summary of the literature in Fig. 5. In our opinion, the main site of dysregulation in RA, is the incomplete phenotype switch from M1 to M2c macrophages, which is reflected by an “indecisive” M2b phenotype which can signal either pro- or anti-inflammatory, depending on signalling from its extracellular environment, but does either ineffectively. This has several knock-on effects, such as the dysregulation of the ECM, which limits satellite cell activation and function, as well as contributing to fibrosis. Both these outcomes then further affect other cell types in a self-reinforcing chronic cascade resulting in the increased fat and collagen deposition, and loss of muscle fibre maintenance observed in rheumatoid cachexia.

In our opinion, there are two main avenues to follow for therapeutic intervention. Firstly, an obvious therapeutic strategy would be to prevent the incomplete M1-M2 transition of macrophages. Secondly, modulation of the RA ECM would not only correct satellite cell functionality, but also improve mature muscle fibre and whole muscle functionality and force output, both of which would significantly contribute to complete repair and return to function. A specific target for this approach would be to reduce the proliferation of fibroblasts, present in the affected muscle as well as the affected joint. However, fibroblasts do not act alone in the development of fibrosis, which is also stimulated by FAPs and macrophages. Of course, given the complexity of the disease and interplay of contributing cell types—especially once properly established—it would be naïve to consider intervention at any one site in isolation.

The fact that rheumatoid cachexia occurs secondary to primary auto-immune disease, and that inflammation itself is a systemic phenomenon rather than a local one, adds complexity to the quest for a therapeutic strategy. However, recent advances in drug delivery systems and nanotechnology may hold promise for delivery of modulating factors into the muscle ECM. Due to the greater presence of macrophages in affected tissues, the proposed use of drugs encapsulated by phagosome-arrested macrophages to deliver treatment to the desired site is a promising one [98]. Additionally, the use of macrophages to deliver satellite cells to the affected muscle holds promise in promoting skeletal muscle regeneration [99].

### Are current treatment strategies failing?

Perhaps due to the complexity of RA as a condition (and even the lack of clarity regarding its precise aetiological trigger), as well as the limitations to be overcome in the development of targeted therapeutic approaches, therapeutic strategies for RA still have ample room for improvement. The main therapeutic goal of current treatment strategies is to induce sustained clinical remission or to maintain a low-inflammatory activity of the disease if remission is not possible [100]. This section will give an overview of the current treatment strategies and briefly outline which of these strategies shows potential in terms of targeting the sites we have identified, in the context of delaying or limiting the extent of rheumatoid cachexia.

Popular treatment options for RA vary from the use of non-steroidal anti-inflammatory drugs (NSAIDs), glucocorticoids, disease-modifying anti-rheumatic drugs (DMARDs), and more recently, biological-response mediators [101, 102]. The treat-to-target (T2T) approach has been found to be more effective than specific treatment strategies and incorporates aspects such as tight control and monitoring of disease and its biological side effects, and therapeutic adjustments when set targets are not met [103]. NSAIDs and glucocorticoids are considered a first-line therapy and act to rapidly reduce pain and swelling in the affected joints by transiently reducing the inflammation present. The use of both NSAIDs and glucocorticoids are only recommended as short-term treatment or as bridge therapy while the DMARDs gain sufficient effectivity, as they neither slow the progression of RA, nor have disease modifying effects and are linked to a large number of adverse effects [104, 105]. Currently, the drugs of choice for the treatment of RA are DMARDs [105]. DMARDs are slower acting compounds that improve symptoms and slow progression of RA. DMARDs are split into two, namely synthetic DMARDS and biological DMARDS [103, 105]. The most commonly used DMARD is methotrexate, which has several mechanisms of action. It has the ability to inhibit the proliferation of cells, including inflammatory-cell mediators and lymphocytes, as well as to reduce TNF-α and IL-1β expression, hence its anti-inflammatory effect [106, 107]. Studies on methotrexate have also indicated a reduction in the accumulation of toxic compounds such as polyamines that contribute to tissue damage [108] as well as a reduction in reactive oxygen groups in the synoviocytes obtained from RA patients [108]. The European League Against Rheumatism (EULAR) still recommends that immediate treatment with DMARDs should occur upon RA diagnosis, and despite the fact that newer therapeutics have been developed, methotrexate should be the first treatment option [103]. Whether methotrexate would be beneficial in rheumatoid cachexia is unknown. Investigation into the effects of methotrexate on satellite cells and skeletal muscle is lacking in the RA model and treatment was unable to shift macrophage phenotype to M2 macrophages once polarised to the M1 phenotype in an in vitro model [109]. The effect of methotrexate on fibrosis is also less desirable—the drug were demonstrated to be pro-fibrotic in both liver cells in vitro [110] and in liver tissue of RA patients [111]. While this treatment may be beneficial and used as a first option therapy for RA, it is not necessarily beneficial in treating or preventing the development of secondary symptoms such as rheumatoid cachexia.

In terms of biological-response modifiers, scientists have largely focused on suppression of systemic inflammation, developing therapies targeting specific soluble or cell-surface molecules with the use of monoclonal antibodies and receptor constructs. One of the most successful targets is TNF-α inhibition (adalimumab, etanercept, and infliximab)—these medicines are often used in conjunction with methotrexate [103, 112, 113]. Etanercept targets TNF-α type II receptor-IgG1 fusion protein while infliximab and adalimumab are specific TNF-α monoclonal antibodies. The use of infliximab with methotrexate significantly improved symptoms of RA including joint swelling, pain, joint damage progression, and CRP concentration compared to that of methotrexate alone in a study assessing 428 patients with active RA [114]. However, in this study there was still a high rate of adverse events, with the most common being infections—which likely resulted from the blanket approach to immune suppression. The use of anti-TNF-α (adalimumab) treatment reduced the diseased joint structural progression compared to methotrexate, which was further reduced by use of the both drugs in combination in a study on 799 patients with active early RA (< 3 years since diagnosis) [115]. However, there are still patients who do not respond to the anti-TNF-α therapy. Additional biologics treatment approaches include the blocking of IL-6 through anti-IL-6-receptor monoclonal antibodies [116], anti-B-cell therapy [117], and down-regulation of T-cell activation through the modulation of the co-stimulatory signal necessary for activation [118]. Due to many cytokine receptors signalling via Janus kinases (JAKs), another more recent treatment strategy involves JAK inhibitors. JAK inhibitors can be used to determine the effect of inhibition of several cytokines; these include IL-6, GM-CSF, type 1 interferons, IL-7, IL-15, and IL-21 [119]. While still in the earlier stages of investigation, tofacitinib/methotrexate combination treatment has demonstrated suppressive effects on T-cells, B-cells and fibroblast-like synoviocytes, as well as reductions in MMPs in 15 RA patients compared methotrexate-treated controls [120].

Despite the advancement of therapeutic strategies to reduce RA disease progression and/or manage pain, not all of these are beneficial in the context of muscle health. While the cachexic effects of the drugs have not been sufficiently investigated in RA patients specifically, their commonly known pathways can be extrapolated to the RA context. For example, glucocorticoids decrease muscle anabolism and increase muscle catabolism through different pathways, including the myostatin pathway, the IGF-1-PI3K-Akt pathway, and the NF-κB pathway [121]—thus, in particular patients showing significant RA cachexia, this treatment should be avoided or at least paired with therapy that may counter these undesired outcomes. In contrast, the use of DMARDs may also limit sarcopenia due to its inhibition of cytokines such as TNF-α and IL-6. However, DMARDS have been linked to increased body weight [122]—given the negative effects of muscle fat deposition commonly seen in rheumatoid cachexia, this problem may be exacerbated by these treatments.

Research on specific therapeutic strategies targeting rheumatoid cachexia are limited. Current therapeutic strategies to target cachexia include increasing physical activity, especially in the form of resistance exercise, and dietary alterations, such as a Mediterranean diet supplemented with omega 3 and vitamin D [123] and antioxidant supplements [124]. Low IGF-1 expression is associated with lower appendicular lean muscle index and muscle CSA in RA patients [125]. The use of IGF-1 treatment increased body weight and gastrocnemius weight, as well as inhibiting the CIA-induced increase in atrogin-1 and MuRF1 expression in rodents [35]. However, IGF-1 is associated with increased cancer risk [126]. Treatment with a peroxisome proliferator-activated receptor-α (PPARα) agonist had similar effects, attenuating the decrease in gastrocnemius weight and fast-twitch myofibre size, and preventing the arthritis-induced increase in atrogin-1 and MuRF1 expression in a rodent CIA model [126]. In addition to positive effects in skeletal muscle, PPAR-α also has anti-inflammatory effects and studies have determined that treatment with PPAR-α agonist resulted in reduced oedema and arthritis score in arthritic rodents [127]. However, the use of PPAR agonists are not an option due to their many side effects, including congestive heart failure, bone fractures, liver disease and myopathy [128].

A number of studies have explored the idea of using physical activity and exercise training to improve muscle strength and mobility in RA patients. Regular range of motion exercise over a 2 year period significantly improved strength, disease activity, and physical function, which was further improved in RA patients undergoing 2 years of dynamic strength training [129]. A 5-week rehabilitation exercise program improved quadriceps strength and activation, and reduced subjective disability without exacerbating disease activity, improvements which were maintained at a 6 month follow up session [130]. Twelve weeks of moderate intensity pool exercise, comprising of a variety of strength, endurance and flexibility exercises, improved grip force and muscle function despite not improving aerobic capacity in RA patients [131]. During a 6-week hand exercise programme, RA patients responded similarly to healthy controls, with an increase in hand force measurements, hand function, and increased CSA in the extensor digitorum communis muscle [132]. While the majority of these studies have demonstrated beneficial effects on muscle strength and function, few of them report on the cachexia outcomes.

## Conclusion

This review highlights the complexity and multi-directionality of cellular interplay in RA cachexia progression. More investigation is required to determine the specific interaction between the cell types mentioned, as limited research has focused on these pathways in terms of rheumatoid cachexia. Review of current treatment strategies illustrates that blanket-approach systemic anti-inflammatory intervention is effective to a degree in RA, but not without side effects or missing the impact on cachexia. The severe impact of rheumatoid cachexia on long term patient independence highlights the importance of addressing not only the primary auto-immune disease, but also its secondary debilitating conditions. Further research is required to develop a more specifically targeted treatment approach, potentially making use of more specific, controlled delivery systems that may be incorporated into T2T approaches to limit rheumatoid cachexia development.

## Supplementary Information

**Additional file 1.** Protocol for immunofluorescence staining, used in preparation of Figure 1.

## Data Availability

Not applicable.

## Abbreviations

AIA: Adjuvant-induced arthritis
AP-1: Activator protein-1
CCL: Chemokine (C–C motif) ligand
CCL2: Monocyte chemoattractant protein-1
CCL3: Macrophage inflammatory protein 1α
CHD: Chronic heart disease
CIA: Collagen-induced arthritis
COPD: Chronic obstructive pulmonary disorder
CRP: C-reactive protein
CSA: Cross sectional area
DMARDs: Disease-modifying anti-rheumatic drugs
ECM: Extracellular matrix
EDL: Extensor digitorum longus
EMT: Epithelial to mesenchymal transition
EULAR: European league against rheumatism
FAPs: Fibro-adipogenic progenitor cells
GM-CSF: Granulocyte–macrophage colony-stimulating factor
IFN-γ: Interferon-γ
IGF: Insulin-like growth factor
IL: Interleukin
IRF: Interferon regulatory factor
JAK: Janus kinase
LPS: Lipopolysaccharide
MAPK: Mitogen-activated protein kinase
miRNA: Micro-RNA
MMP: Matrix metalloproteinase
Murf1: Muscle ring finger-1
MyoD: Myoblast determination protein 1
NF-κB: Nuclear factor kappa-light-chain enhancer of activated B cells
NLRP3: NOD-, LRR- and pyrin domain-containing protein 3
NSAIDs: Non-steroidal anti-inflammatory drugs
PAI-1: Plasminogen activator inhibitor type-1
Pax7: Paired box protein-7
PCNA: Proliferating cell nuclear antigen
PI3k/Akt: Phosphatidylinositol 3-kinase (PI3K)/protein kinase B (AKT)
PPAR-γ: Peroxisome proliferator-activated receptor gamma
QCT: Quantitative computer tomography
RA: Rheumatoid arthritis
RM: Rheumatoid myositis
Rrbp1: Ribosomal binding protein 1
T2T: Treat-to-target
TA: Tibialis anterior
TGF-β: Transforming growth factor-β
TIMP: Tissue inhibitors of metalloproteinase
TNF-α: Tumour necrosis factor-α
