# Supplementary material for: Rheumatoid cachexia: the underappreciated role of myoblast, macrophage and fibroblast interplay in the skeletal muscle niche
Source: J Biomed Sci. 2021 Mar 3;28:15. doi: 10.1186/s12929-021-00714-w (PMC7931607; doi:10.1186/s12929-021-00714-w)
Supplement: Supplementary file 1 — Additional file 1. Protocol for immunofluorescence staining, used in preparation of Figure 1. [file 12929_2021_714_MOESM1_ESM.docx]

Supplementary material

Figure 1 method

RA rodent study carried out as described in Oyenihi et al., 2019 [35]. Skeletal muscle tissue was donated for use in preparation of this manuscript.

Immunofluorescence staining for the development of Figure 1

In order to design figure 1, three immunofluorescent stains were performed on serial sections of gastrocnemius tissue and imaged on a confocal microscope (Carl Zeiss LSM 780, Zeiss, Germany). Briefly, subsequent slides were stained with Pax7 (satellite cells; ab 528428, Dev. Studies Hybridoma Bank, USA), F4/80 (macrophages; sc377009, Santa Cruz Biotechnology, USA), and α-smooth muscle actin (fibroblasts; A2547, Sigma-Aldrich, Germany) after fixation in 4% paraformaldehyde and blocking in 5% donkey serum. Appropriate secondary antibodies were added, followed by 488-conjugated wheat germ agglutinin for visualisation of muscle fiber borders. Finally, Hoechst was added to visualise nuclei and coverslips mounted with fluorescent mounting media. Images were analysed using ImageJ (Wayne Rasband) in which individual cells and fibers were counted and compared.
